# The value of quantitative patient preferences in regulatory benefit-risk assessment

**DOI:** 10.3402/jmahp.v2.22761

**Published:** 2014-04-01

**Authors:** Mart oude Egbrink, Maarten IJzerman

**Affiliations:** Department Health Technology and Services Research, School of Management and Governance, University of Twente, Enschede, The Netherlands

**Keywords:** patient involvement, preferences, benefit-risk assessment, European Medicines Agency

## Abstract

Quantitative patient preferences are a method to involve patients in regulatory benefit-risk assessment. Assuming preferences can be elicited, there might be multiple advantages to their use. Legal, methodological and procedural issues do however imply that preferences are currently at most part of the solution on how to best involve patients in regulatory decision making. Progress is recently made on these issues.

In recent years, a fundamental discussion has taken place on how market approval and reimbursement authorities can better involve patients and patients’ perspectives (1). Typically, the argument to include the patients’ voice is that the quality of the decision increases because patients provide valuable experiential knowledge about living with a condition (2, 3). Involving patients can be important because they may provide additional evidence about the severity for them of particular adverse events and, hence, their tolerance to these risks (4). Further, it promotes transparency and legitimacy because the ones affected by the decisions are involved in the decision-making process. This may lead to a higher public acceptance of market authorization decisions (2, 5). These arguments are consistent with the emerging need for patient-based health technology assessment (HTA) in which patients are empowered to take control over their own health (6).

Patients can be involved by different means. Typically, a distinction is made among communication, consultation, and participation for public engagement mechanisms (7). This distinction differentiates on the basis of information flow between the public involved and the sponsor. Communication implies that stakeholders are only informed by the organization commissioning the initiative. In consultation, the information flows in the opposite direction – from the stakeholders to the sponsor, on the latter's initiative. Participation is about the exchange of information. This suggests that a dialogue can take place in which opinions can be shaped (7). Recently, this categorization has also been applied in research on patient involvement in HTA (2, 3, 5, 8).

In addition, two complementary approaches for patient involvement can be distinguished – that of direct representation through participation in a committee or advisory group or an indirect approach using methods that allow the study of a patient's preferences regarding treatment characteristics (e.g., benefits and risk) as input for the decision maker (2). Obviously, these are not mutually exclusive. Preference elicitation methods can be considered a form of consultation for benefit-risk assessments (7). Here, a distinction can be made between qualitative techniques, which are useful for gaining in-depth knowledge about the value of a specific drug (2), and quantitative techniques (3). Quantitative techniques do elicit a patient's preference for alternative options by comparing multiple attributes such as benefit and risk profiles presenting benefit-risk trade-offs (9).

## Benefit–risk assessment at the European Medicines Agency

In the EU, the European Medicines Agency (EMA) is responsible for market authorization and pharmacovigilance on behalf of the member states (4). In market authorization, they balance the benefits and harms of a new therapeutic (4, 10). While making these judgments, the EMA considers the patient perspective because this enriches regulatory decisions (11), gives the public more confidence in the outcomes, and ultimately contributes to the quality of the decisions made. Although overall involvement of patients in EMA activities has been growing considerably in the past years in terms of number of patients and number of occasions (12), the degree of involvement specifically in benefit–risk judgments is not clear. Currently, the EMA is working on a policy document describing how patients are and should be involved in benefit–risk assessments. This policy document ought to be integrated in a new framework on interaction with patients by the end of the year 2013 (11, 12). Although it is not likely that quantitative patient preferences will already be part of this framework, we suggest including them in the future.

## Preference elicitation methods

There have been a growing number of patient preference studies over the past 30 years (13, 14). Preference elicitation, or more formally, stated preference studies for measuring quantitative preferences are to be distinguished from revealed preferences because elicited preferences are obtained in experimental studies offering choices. For stated preferences, there are two main categories – those that estimate the marginal utility for a specific or combination of attributes and those that estimate the monetary value of an intervention. Conjoint analysis or discrete choice experiments and multiple-criteria decision analysis (MCDA) usually involves methods that provide a relative weight or marginal utility for a given set of attributes. Contingent valuation or willingness to pay (WTP) studies typically determine the monetary value of an intervention (15–18).

The EMA has already identified conjoint analysis as a method that could help regulators in judging trade-offs between favorable and unfavorable effects (19). Using conjoint analysis, different attributes of a drug can be considered jointly. Patients are asked to express their preferred option in a set of two or three treatment profiles where attributes (e.g., specific benefits and/or risks) are varied. This allows the estimation of the importance or preference for the individual attributes as well as for the treatment scenarios. Outcome measures are, for instance, the (relative) importance of different attributes and the maximum acceptable risk patients are willing to accept (20).

MCDA also is a method that allows the elicitation of patients’ weights for the criteria considered. One such example is the Analytic Hierarchy Process (AHP) (16, 21). The AHP has been used to demonstrate that patient relevant endpoints can be prioritized and weighted by decomposing a decision problem into multiple criteria and by then applying pair wise comparisons of the alternatives on the criteria (22). It is beyond the scope of this short communication to further discuss methods, and the reader is referred to other studies (16, 23).

## Elicited patient preferences in benefit–risk assessment

Assuming preferences can be elicited, they could potentially play a role in regulatory decision making. Quantifying patient preferences yields relative importance weights for benefits and risks and thus facilitates direct comparisons for drug approval decisions. This allows the use of preferences for assisting in the interpretation of clinical evidence on benefits and harms obtained from randomized controlled trials, but it also helps in comparing the patients’ risk tolerance with that of regulators (9). Preferences also provide a means for transparent (17) and consistent inclusion of patients’ perspectives in benefit–risk decisions (15). Consequently, preference data might help patients manage the complex information in assessments (24). This could empower patients when involved in appraisal committees such as EMA's Committee for Medicinal Products for Human Use (CHMP). Another advantage of elicited preferences is that it could, depending on the use, improve representativeness because problems such as lack of clarity about who to involve and the absence of adequate representation can be avoided (5). Further, current benefit–risk assessments are mainly focused on objective clinical evidence (24). By using elicited preferences, it may be possible to also obtain a (patient) perspective on factors that are typically not captured in clinical trials (5).

In previous meetings with the EMA, it was acknowledged that patient preferences could indeed help improve transparency and communication of benefit–risk assessments. Yet several legal, methodological, and procedural issues were identified that need to be discussed before considering the use of patient preferences in regulatory decision making.

Legal issues relate to commissioning responsibility for collecting patient preferences, the reliability of preference data, and to the consequences of potential biases that may occur. In addition, there are some methodological issues to consider. For instance, innumeracy, the use of heuristics, variation among subgroups, inert or flexible preferences, measurement error, and hypothetical bias are mentioned (4, 17, 18, 25). Different methods for preference elicitation are being used, and a clear guidance on the appropriate use is required. One other field of current development is that of approaches for using preferences to assist in the evaluation of clinical evidence (for example, in a quantitative model that allows integration of preference and performance data) (26).

Another field of current investigation relates to the best way to use patient preferences in the regulatory process. For instance, how can patient organizations involved in EMA best use quantitative patient preferences, and do these patient organizations expect to benefit from quantitative patient preferences to express their perspective in the decision-making process?

## Future perspective

Current research on patient involvement at the EMA mainly relates to deliberation on the benefit–risk balance in market authorization. Although this is one important role for patient involvement, patient views may be important in all stages (6) – from early drug development to market authorization and reimbursement. Eventually, this may be beneficial for all stakeholders involved, including industry (9). At the same time, preferences can also be used in pharmacovigilance because the benefit–risk balance may change due to new evidence, for instance, on adverse effects (9).

The multiple aspects and levels at which patients need to be involved, together with the possible disadvantages of preference elicitation methods, implies that preferences are at most just part of the solution. Recent developments in stated preference methodology and an evolving consensus on best practices are required to address legal and methodological concerns in regulatory decision making. One such development is the International Society for Pharmacoeconomics and Outcomes Research (ISPOR) checklist for the application of conjoint analysis (15). Besides methodological guidance, lessons could also be drawn from the U.S. Food and Drug Administration, which has experience using patient preferences (27). As these findings become available, a better understanding can be obtained about the potential of preferences as a way of representing patient views and of increasing the quality of the decisions made. However, if we want to achieve a sufficient understanding of the potential of patient preferences, more research is needed that should not only focus on the use of and methodological aspects of preferences but also on preferences as part of the arrangement of changes in health care.
